# Short-Term Changes in Segmental Multi-Frequency Bioelectrical Impedance Measurements Following Water and Banana Intake: A Fixed-Order Within-Subject Study

**DOI:** 10.7150/ijms.134700

**Published:** 2026-10-02

**Authors:** Erika Čermáková, Monika Kunzová, Jana Olejníčková, Petr Loskot, Martin Forejt

**Affiliations:** 1 Department of Public Health, Faculty of Medicine, Masaryk University, Brno, Czech Republic.; 2 Department of Physiology, Faculty of Medicine, Masaryk University, Brno, Czech Republic.; 3 International Clinical Research Center, St Anne's University Hospital, Brno, Czech Republic.

**Keywords:** body composition, segmental multi-frequency bioelectrical impedance analysis, fluid intake, food consumption, percentage of body fat, visceral fat estimate

## Abstract

**Background:**

Bioelectrical impedance analysis (BIA) is widely used for body composition assessment but may be influenced by hydration status and recent food intake. This study evaluated short-term changes in segmental multi-frequency BIA measurements following water and banana intake.

**Methods:**

Ninety-eight healthy adults (54 women, 44 men; 21-33 years) underwent BIA measurements at baseline and at 30, 60, and 90 minutes after consuming 600 mL of water on the first experimental day and 300 g of banana on the second day. The study used a fixed-order within-subject repeated-measures design. Post-ingestion values were analyzed using baseline-adjusted linear mixed-effects models with participant-specific random intercepts. Holm's procedure was applied across the five time × experimental condition/day interaction tests.

**Results:**

Significant time × experimental condition/day interactions were not observed for body fat percentage, total body water percentage, or trunk impedance at 20 or 100 kHz (all unadjusted p ≥ 0.133). A significant interaction was observed for visceral fat area estimate (F(2,194) = 8.532, unadjusted p < 0.001; Holm-adjusted p < 0.005). The largest adjusted between-condition/day difference in visceral fat area estimate was 0.858 cm² at 30 minutes. Because intake condition was fixed by study day, condition effects could not be separated from potential day or order effects.

**Conclusions:**

Under the fixed-order conditions examined, temporal responses were similar between experimental days for most SMF-BIA outcomes, while a small interaction was observed for visceral fat area estimate. These findings require confirmation in randomized, counterbalanced studies including a no-intake control condition.

## Introduction

Bioelectrical impedance analysis (BIA) is currently one of the most commonly used methods for assessing body composition, particularly the proportion of fat and fat-free mass and total body water 1. The assessment of body composition, especially the amount and distribution of fat, is an important indicator of health and is crucial in clinical practice when assessing nutritional status in obesity or other forms of malnutrition, as well as in research focused on body composition 2. Unlike body mass index (BMI), which is based solely on height and weight, BIA allows for a more detailed assessment of body composition by distinguishing between different tissue compartments 1,3. The method is non-invasive, fast, and relatively inexpensive 1,4.

Despite these advantages, the accuracy of BIA measurements depends on adherence to standardised protocols. The method involves passing an alternating electric current through the body, with different tissues offering different levels of resistance and reactance 5. Therefore, the results can be significantly affected by hydration status and the contents of the gastrointestinal tract 1,6. For this reason, it is generally recommended that measurements are performed on an empty stomach, after avoiding food and liquids for a period of time 5.

Although a number of studies have demonstrated the acute influence of prior fluid or food intake on body composition results, the magnitude and direction of this effect varies between studies, and its clinical significance is unclear 7-12. While some studies have reported significant changes in BIA parameters after fluid intake 10,13 or energy-rich meals 7, others have observed no alterations following a typical snack 14.

There is still no uniform, universally accepted consensus on the exact length of fasting prior to BIA 15. For example, the National Institutes of Health previously stated in its Technology Assessment Conference Statement that food and fluid intake can affect BIA values and emphasized the need for standardization of measurement procedures, including controlled measurement conditions and hydration of subjects. It recommends approximately 4 hours of fasting prior to BIA measurement 5. The European Society for Clinical Nutrition and Metabolism (ESPEN) recommends performing BIA after fasting for 2-8 hours, with specific recommendations varying depending on the context of use of the method 16,17. These and other 18 recommendations are based on studies with different measurement protocols and specific conditions, which limits their generalizability and clinical applicability 17,18.

As the recommended fasting period increases, the practical application of BIA becomes less feasible, both in research and in clinical practice. For example, longer fasting periods are undesirable for patients with sarcopenia or malnutrition 17,19.

At the same time, relatively few studies have directly compared short-term changes in BIA measurements following typical amounts of fluid and solid food intake in the same individuals. Much of the available evidence has focused on relatively large fluid loads or energy-rich meals, which may not fully reflect common clinical or field conditions 7,20,21.

From a practical perspective, it remains unclear how BIA-derived parameters vary in the short term following consumption of a typical amount of water or a portion of fruit. Both represent plausible deviations from recommended pre-assessment conditions, and a better understanding of the magnitude of such variation may help inform the interpretation of measurements obtained under less strictly standardized conditions.

The aim of this study was therefore to evaluate short-term changes in SMF-BIA outcomes following consumption of 600 mL of water and 300 g of banana in the same participants using a fixed-order within-subject repeated-measures design.

## Methods

### Subjects

A total of 98 healthy adults (54 women, 44 men) aged 21 to 33 voluntarily participated in this study. The study was approved by the Ethics Committee of the Faculty of Medicine at Masaryk University in Brno. All study participants signed an informed consent form prior to participating in the research. The data obtained from the subjects are anonymous, recorded under a numerical code, and processed without identifying information. The results of the body composition assessment were provided to and explained to the subjects.

### Anthropometric measurements

Basic anthropometric methods were used to determine the anthropometric characteristics of the subjects - measurement of body height and determination of body composition using bioimpedance analysis (BIA). All examinations were performed by professionals trained to work with the given devices. Body height was measured using a SECA 764 height meter (SECA GmbH & Co., Hamburg, Germany), which had an accuracy of 0.1 cm. Body composition was measured using an InBody 230 device (Biospace, Seoul, South Korea), and Lookin'Body 3.0 (Biospace, Seoul, South Korea) software for processing and evaluating body composition data. The InBody 230 device underwent routine annual calibration in accordance with institutional procedures. During the body composition measurement, the subject stood upright on the InBody 230 device, with all eight electrodes placed in pairs on each arm and leg The outcomes analyzed in the present study were trunk impedance at 20 and 100 kHz as directly measured bioelectrical parameters, and body fat percentage, total body water percentage, and visceral fat area estimate as device-derived outcomes. Body weight was re-measured by the integrated scale of the InBody 230 at each measurement time point (baseline, 30, 60, and 90 minutes) and the contemporaneous weight value was used by the device for body composition estimation; baseline weight was not held constant across repeated measurements. To contextualize the potential contribution of contemporaneous body weight to device-derived estimates, changes in measured body weight from baseline were summarized descriptively at 30, 60, and 90 minutes for each experimental day.

In order to ensure measurement accuracy, the subjects were dressed only in underwear or tight-fitting swimsuits (two-piece swimsuits for women). The participants' feet were bare. The subjects did not consume any food or liquids for 4 hours prior to the measurement, avoided strenuous physical activity (e.g., endurance running, swimming, weight training) for 12 hours prior, did not consume alcohol for 2 days prior, and did not take any diuretics for 7 days prior. Before the measurement, the subjects had an empty bladder, were not allowed to eat or drink anything except for fluids and food related to the study during the measurement, and sat quietly in the examination room at a constant temperature of 22°C. Further emptying of the bladder was only allowed after the last measurement, i.e., 90 minutes after consuming food or liquids related to the study. Compliance with pre-measurement restrictions was verified before each experimental session using standardized verbal screening.

### Study procedures

The study used a fixed-order within-subject repeated-measures design in which all participants completed both experimental conditions on two consecutive days. The sequence of conditions was the same for all participants and was neither randomized nor counterbalanced.

On the first experimental day, participants consumed 600 mL of tap water. SMF-BIA measurements were performed immediately before consumption (baseline) and at 30, 60, and 90 minutes after intake. On the second experimental day, participants consumed 300 g of banana, with measurements performed according to the same schedule. Participants were given 5 minutes to consume the assigned water or food.

No no-intake control condition was included. Because water was always administered on the first day and banana on the second day, intake condition was inherently confounded with study day and order. Accordingly, comparisons between conditions cannot distinguish the effects of intake type from potential day- or order-related effects.

### Statistical data analysis

All statistical analyses were performed using IBM SPSS Statistics, version 31.0 (IBM Corp., Armonk, NY, USA). Statistical tests were two-sided, with a significance level of p < 0.05.

No *a priori* sample-size calculation was performed. The analysis included all participants who completed measurements on both experimental days.

Baseline values obtained on the two experimental days were compared using paired-samples t-tests. Because small baseline differences were observed between experimental days, longitudinal analyses were performed using baseline-adjusted linear mixed-effects models. Only post-ingestion measurements obtained at 30, 60, and 90 minutes were included in these models, with the corresponding baseline value for each experimental day entered as a covariate.

Each SMF-BIA outcome was analyzed separately. The models included fixed effects for time (30, 60, and 90 min), experimental condition/day (water vs. banana), and the time × experimental condition/day interaction. A participant-specific random intercept was included to account for repeated observations within individuals, and an unstructured covariance matrix was used to model repeated measurements within each experimental condition/day. Denominator degrees of freedom were estimated using the Satterthwaite method.

Because water was administered on the first experimental day and banana on the second day for all participants, experimental condition was fully confounded with study day and order. Consequently, condition, day, and order effects could not be estimated independently, and the condition effect was interpreted as an experimental condition/day effect rather than as an independent causal effect of intake type.

To account for multiplicity across the five SMF-BIA outcomes, Holm's sequential Bonferroni procedure was applied to the five time × experimental condition/day interaction tests. Baseline-adjusted estimated marginal means (EMMs) with 95% confidence intervals (CIs) were reported to describe the magnitude and precision of the observed differences. Model adequacy was assessed by inspection of model convergence and residual distributions.

## Results

### Participants and baseline comparisons

A total of 98 participants (54 women and 44 men) completed both experimental days and all repeated measurements. Measurements were obtained at baseline and at 30, 60, and 90 minutes after consumption of 600 mL of water on the first experimental day and 300 g of banana on the second experimental day. Baseline demographic and SMF-BIA characteristics stratified by sex are presented in Supplementary Table S1.

Baseline values differed between experimental days for the SMF-BIA-derived visceral fat area estimate (mean difference, water minus banana: 1.352 cm²; p = 0.002), body fat percentage (0.277 percentage points; p = 0.017), and total body water percentage (-0.204 percentage points; p = 0.016). Baseline trunk impedance at 20 and 100 kHz did not differ significantly between experimental days (both p > 0.05; Supplementary Table S2).

Measured body weight increased from baseline by 0.462 ± 0.325 kg, 0.378 ± 0.288 kg, and 0.333 ± 0.361 kg at 30, 60, and 90 minutes, respectively, on the water day. Corresponding increases on the banana day were 0.176 ± 0.288 kg, 0.175 ± 0.268 kg, and 0.143 ± 0.275 kg.

### Baseline-adjusted mixed-effects analysis

Baseline-adjusted linear mixed-effects models were used to evaluate post-ingestion measurements at 30, 60, and 90 minutes (Table 1). A significant time × experimental condition/day interaction was observed for the SMF-BIA-derived visceral fat area estimate (F(2, 194) = 8.532, unadjusted p < 0.001), and this interaction remained significant after Holm correction for the five interaction tests (adjusted p < 0.005).

No significant time × experimental condition/day interactions were observed for body fat percentage (F(2, 194) = 0.852, p = 0.428), total body water percentage (F(2, 194) = 0.879, p = 0.417), trunk impedance at 20 kHz (F(2, 194) = 0.867, p = 0.422), or trunk impedance at 100 kHz (F(2, 194) = 2.036, p = 0.133). None of these interactions was significant after adjustment for multiple testing.

For the visceral fat area estimate, baseline-adjusted estimated marginal means differed most between experimental days/conditions at 30 minutes (56.140 cm² for the water day vs. 55.282 cm² for the banana day), corresponding to an adjusted difference of 0.858 cm². The difference was 0.156 cm² at 60 minutes and -0.275 cm² at 90 minutes (Table 2).

Main effects of time and experimental condition/day are presented in Table 1. Significant time effects were observed for the visceral fat area estimate, body fat percentage, and total body water percentage, whereas no significant time effects were observed for trunk impedance at 20 or 100 kHz. Significant experimental condition/day effects were observed for body fat percentage, total body water percentage, and trunk impedance at both frequencies, but not for the visceral fat area estimate. Because experimental condition was fixed by study day, these condition/day effects cannot be separated from potential day or order effects and were not interpreted as independent effects of water versus banana intake.

Baseline-adjusted estimated marginal means with 95% confidence intervals are presented in Table 2. Across outcomes, between-day/condition differences were small in absolute magnitude.

## Discussion

In this fixed-order within-subject study, baseline-adjusted analyses showed that the temporal response over 30, 60, and 90 minutes did not differ between experimental conditions/days for body fat percentage, total body water percentage, or trunk impedance at 20 and 100 kHz. A significant time × experimental condition/day interaction was observed for visceral fat area estimate and remained significant after correction for multiple testing. However, the corresponding between-condition/day differences were small in absolute magnitude, with the largest adjusted difference being less than 1 cm². Because water was always administered on the first day and banana on the second, the observed effects cannot be attributed independently to intake type.

The available literature on the acute effects of food and fluid intake on BIA measurements remains inconsistent 7,9,10,13,14,20,22,23. Several studies have reported statistically significant changes in impedance or BIA-derived body composition parameters following acute fluid or food intake 7,9,10,13,20,22, whereas others have found little or no measurable effect after more typical food or fluid consumption 14,23. Differences among studies may reflect variation in the amount and composition of intake, timing of post-ingestion measurements, characteristics of the study population, and the BIA technology used. In addition, statistically detectable changes in BIA-derived measures do not necessarily indicate a meaningful alteration in body composition 9,13,24.

The present findings add to this evidence by showing that most baseline-adjusted temporal responses were similar between the two experimental days. Although visceral fat area estimate showed a statistically significant interaction, the adjusted differences between conditions/days ranged from -0.275 to 0.858 cm² across the post-ingestion measurements. This distinction between statistical detectability and practical importance is relevant when interpreting small changes in body-composition measurements 25,26. Nevertheless, because no predefined threshold for clinically meaningful change was available for the present device and outcomes, the clinical relevance of these small differences cannot be established directly.

From a practical perspective, the results suggest that the short-term variation observed after consumption of 600 mL of water or 300 g of banana was limited under the specific conditions examined. However, the present design does not provide sufficient evidence to support relaxation of existing pre-measurement standardization recommendations 5,16. In particular, the absence of a no-intake control condition prevents separation of intake-related changes from variation associated with repeated measurements or the passage of time.

The small short-term variations observed are compatible with the bioelectrical principles underlying BIA. BIA estimates are derived from the electrical properties of body tissues, which are influenced by their water and electrolyte content 15,27. In multi-frequency BIA, impedance is measured at different current frequencies, which differ in their interaction with biological tissues and fluid compartments 27,28. Raw impedance measurements should therefore be distinguished from device-derived body composition estimates, which are generated using device-specific prediction algorithms. Short-term fluctuations in parameters such as body fat percentage, total body water, or visceral fat area estimate should consequently not be interpreted as direct evidence of acute changes in the corresponding tissues. Because body weight was re-measured at each time point, the ingested mass contributed directly to the contemporaneous scale measurement. The observed increase in measured body weight was greatest at 30 minutes, averaging 0.462 kg on the water day and 0.176 kg on the banana day, and decreased thereafter. Short-term variation in device-derived body composition estimates should therefore be interpreted as potentially reflecting a combination of changes in measured body mass and electrical impedance rather than physiological changes in the corresponding tissues alone. The relative contribution of these components cannot be quantified from the present data.

The statistically significant interaction observed for the visceral fat area estimate should also be interpreted in the context of measurement precision. Lee et al., using the same InBody 230 model, reported a test-retest coefficient of variation of 0.24% for visceral fat area estimation under controlled conditions. However, this repeatability metric does not represent a threshold for clinically meaningful change. In a repeated-measures study using the related InBody 770 platform, estimated variability in visceral fat area was approximately 1.1 cm² between repeated tests and 2.5 cm² between days. Against this context, the maximal adjusted between-condition difference of 0.858 cm² observed in the present study was small in absolute magnitude and may overlap with short-term measurement or biological variability. Nevertheless, because precision estimates are device- and protocol-specific, the observed difference cannot be definitively classified as measurement noise 24, 29.

Several strengths of the study should be noted. All 98 participants completed both experimental days and repeated measurements at four time points, reducing the influence of between-person variability. The analysis incorporated baseline values directly and used linear mixed-effects models to account for the repeated-measures structure of the data. In addition, multiplicity across the five interaction tests was addressed using Holm's procedure, and adjusted estimates were reported with 95% confidence intervals to describe the precision of the observed effects.

The study also has important limitations. First, the sequence of experimental conditions was fixed, with water always administered on day 1 and banana on day 2. Consequently, intake condition was completely confounded with study day and order. Future crossover studies should therefore use randomized, counterbalanced block allocation of condition sequences, together with an appropriate washout interval and identical pre-measurement standardization across visits, to minimize carryover, order, and day-to-day effects. Second, no no-intake time-control condition was included. Consequently, the observed time effects cannot be attributed specifically to ingestion and may also reflect normal diurnal fluid redistribution, postural stabilization during prolonged sitting, repeated-measurement variability, or instrument drift. Inclusion of a resting no-intake control condition under otherwise identical conditions would allow these sources of temporal variation to be distinguished from ingestion-related effects. Third, no a priori sample-size calculation was performed. The follow-up period was limited to 90 minutes, and the study included only young healthy adults measured using a single SMF-BIA device. These factors limit generalizability to other populations, devices, intake amounts, and longer postprandial periods. Finally, although the observed differences were small in absolute magnitude, no predefined clinical or device-specific threshold was available to determine whether they exceeded expected measurement variability.

## Conclusion

In this fixed-order within-subject study, baseline-adjusted temporal responses following consumption of 600 mL of water or 300 g of banana were similar for most SMF-BIA outcomes over 90 minutes. A significant time × experimental condition/day interaction was observed for visceral fat area estimate, although the absolute differences were small. Because intake condition was confounded with study day and order and no no-intake control condition was included, the observed differences cannot be attributed specifically to water or banana consumption. These findings should therefore be interpreted cautiously and confirmed in randomized, counterbalanced studies including a no-intake control condition.

## Supplementary Material

Supplementary tables.

## Figures and Tables

**Table 1:** Baseline-adjusted linear mixed-effects models for post-ingestion SMF-BIA outcomes

Outcome	Effect	F (df1, df2)	p-value	Holm-adjusted p*
Visceral fat area estimate (cm²)	Time	F(2, 194.000) = 7.742	<0.001	—
	Condition/day	F(1, 90.928) = 0.969	0.327	—
	Time × Condition/day	F(2, 194.000) = 8.532	<0.001	<0.005
Body fat (%)	Time	F(2, 194.000) = 8.093	<0.001	—
	Condition/day	F(1, 88.182) = 10.676	0.002	—
	Time × Condition/day	F(2, 194.000) = 0.852	0.428	1.000
Total body water (%)	Time	F(2, 194.000) = 7.713	<0.001	—
	Condition/day	F(1, 193.193) = 11.154	0.001	—
	Time × Condition/day	F(2, 194.000) = 0.879	0.417	1.000
Trunk impedance 20 kHz (Ω)	Time	F(2, 194.000) = 2.898	0.058	—
	Condition/day	F(1, 95.254) = 7.644	0.007	—
	Time × Condition/day	F(2, 194.000) = 0.867	0.422	1.000
Trunk impedance 100 kHz (Ω)	Time	F(2, 194.000) = 2.070	0.129	—
	Condition/day	F(1, 100.195) = 9.602	0.003	—
	Time × Condition/day	F(2, 194.000) = 2.036	0.133	0.532

*Holm-adjusted p-values were calculated across the five Time × Condition/day interaction tests; p-values for other model terms are unadjusted. Linear mixed-effects models were fitted to post-ingestion measurements obtained at 30, 60, and 90 min, with the corresponding baseline value included as a covariate. Models included fixed effects for Time, experimental Condition/day, and their interaction, a participant-specific random intercept, and an unstructured covariance matrix for repeated measurements within each experimental condition/day. Denominator degrees of freedom were estimated using the Satterthwaite method. Because water was always administered on day 1 and banana on day 2, Condition is confounded with study day and order and cannot be interpreted independently as an intake effect.

**Table 2:** Baseline-adjusted estimated marginal means of post-ingestion SMF-BIA outcomes

Outcome	Time (min)	Water, EMM (95% CI)	Banana, EMM (95% CI)
Visceral fat area estimate (cm²)	30	56.140 (55.739-56.541)	55.282 (54.881-55.683)
	60	55.345 (54.881-55.810)	55.189 (54.725-55.654)
	90	55.095 (54.585-55.605)	55.370 (54.860-55.880)
Body fat (%)	30	22.765 (22.600-22.930)	22.450 (22.285-22.616)
	60	22.605 (22.405-22.805)	22.181 (21.981-22.381)
	90	22.696 (22.497-22.894)	22.250 (22.052-22.449)
Total body water (%)	30	56.602 (56.483-56.721)	56.841 (56.722-56.960)
	60	56.725 (56.576-56.873)	57.030 (56.881-57.178)
	90	56.649 (56.503-56.796)	56.992 (56.846-57.138)
Trunk impedance 20 kHz (Ω)	30	26.102 (25.955-26.249)	25.908 (25.761-26.055)
	60	26.059 (25.908-26.210)	25.759 (25.608-25.910)
	90	26.151 (25.983-26.320)	25.843 (25.674-26.011)
Trunk impedance 100 kHz (Ω)	30	22.343 (22.212-22.474)	22.204 (22.073-22.335)
	60	22.365 (22.234-22.496)	22.054 (21.922-22.185)
	90	22.430 (22.284-22.577)	22.136 (21.990-22.283)

* Values are baseline-adjusted estimated marginal means (EMMs) with 95% confidence intervals derived from the linear mixed-effects models described in Table 1. EMMs were evaluated at the mean baseline value of the corresponding outcome.

## Abbreviations

BIA: bioelectrical impedance analysis
BMI: body mass index
CI: confidence interval
EMM: estimated marginal mean
ESPEN: European Society for Clinical Nutrition and Metabolism
SMF-BIA: segmental multi-frequency bioelectrical impedance analysis
