# Supplementary material for: Short-Term Changes in Segmental Multi-Frequency Bioelectrical Impedance Measurements Following Water and Banana Intake: A Fixed-Order Within-Subject Study
Source: Int J Med Sci. 2026 Oct 2;23(11):3547–53. doi: 10.7150/ijms.134700 (PMC13685467; doi:10.7150/ijms.134700)
Supplement: Supplementary file 1 [file ijmsv23p3547s1.pdf]

Supplementary Tables

Supplementary Table S1. Baseline demographic and SMF-BIA characteristics of study participants stratified by sex

Variable	Women (n = 54)	Men (n = 44)	Total (n = 98)
Age (years)	24.31 ± 1.45	24.68 ± 2.46	24.48 ± 1.97
Body weight (kg)	60.40 ± 8.07	77.50 ± 9.78	68.08 ± 12.29
Body fat (%)	26.35 ± 6.47	17.75 ± 6.42	22.49 ± 7.72
Total body water (%)	53.98 ± 4.83	60.28 ± 4.74	56.81 ± 5.71
Trunk impedance, 20 kHz (Ω)	27.87 ± 2.33	24.58 ± 2.24	26.39 ± 2.81
Trunk impedance, 100 kHz (Ω)	24.31 ± 2.22	20.49 ± 2.17	22.59 ± 2.90
Visceral fat area estimate (cm^2)	46.09 ± 20.22	66.19 ± 24.16	55.11 ± 24.15

*Values are presented as mean ± SD. Baseline values correspond to measurements obtained before intake on the first experimental day. SMF-BIA, segmental multi-frequency bioelectrical impedance analysis. Height and BMI were not retained in the archived analytical dataset and are therefore not reported.

Supplementary Table S2. Baseline comparisons between experimental days

Variable	Mean difference	95% CI	t (df)	p-value
Visceral fat area estimate (cm^2)	1.352	0.497 to 2.207	t(97) = 3.138	0.002
Body fat (%)	0.277	0.050 to 0.503	t(97) = 2.421	0.017
Total body water (%)	−0.204	−0.369 to −0.039	t(97) = −2.455	0.016
Trunk impedance 20 kHz (Ω)	0.104	−0.100 to 0.308	t(97) = 1.016	0.312
Trunk impedance 100 kHz (Ω)	0.078	−0.105 to 0.260	t(97) = 0.847	0.399

*Baseline values between experimental days were compared using paired-samples t-tests (n = 98). Mean differences are expressed as water day minus banana day. Two-sided p-values are reported. Because experimental condition was fixed by study day, these comparisons represent differences between experimental condition/days and cannot distinguish intake, day, or order effects.
